# Accelerated water harvesting from vapor via a bio-inspired hydrogel pattern

**DOI:** 10.1093/nsr/nwae067

**Published:** 2024-02-23

**Authors:** Lianbin Zhang, Peng Wang

**Affiliations:** State Key Laboratory of Material Processing and Die & Mould Technology, Key Laboratory of Materials Chemistry for Energy Conversion and Storage of Ministry of Education, School of Chemistry and Chemical Engineering, Huazhong University of Science and Technology (HUST), China; School of Environmental Science and Engineering, Sun Yat-sen University, China

Water and energy sustainability are integral parts of resource sustainability of the world. Due to water molecules’ superior capability of serving as both hydrogen-bond donor and acceptor, phase change of water, especially between liquid and vapor, underpins global water cycles, facilitates energy transformation and transfer, and thus helps shape the unique local weather and global climate patterns. In recent years, research attention towards water vapor condensation has been re-ignited for the purpose of water production and/or heat management. In particular, within many emerging schemes of water production from conventional resources that involve water evaporation, e.g. solar distillation, membrane distillation, and sorption-based atmospheric water harvesting, condensation is the last step and can be the rate-limiting factor of water production [1].

It is agreed that, in the course of condensation, fast droplet growth and shedding on condensing surfaces are essential in order to promote water production kinetics. However, there has long existed a dilemma that hydrophilic surfaces are thermodynamically favorable for nucleation but unproductively suffer from surface flooding, making a sustainable condensation unachievable [2]. On the other hand, there have been reports using amphiphilic patterns to reduce droplet pinning and to bring about random droplet cohesion, but as expected, many of them ended up with insufficient driving forces for regional droplet shedding [3]. Ideally, a smooth droplet shedding calls for controlled and stable droplet movement which in turn is enabled by controlled liquid dynamic behaviors, including but not limited to, full bounce, asymmetrical spreading, and directional transport [4]. In seeking solutions, researchers often turn to nature for inspiration.

**Figure 1.**
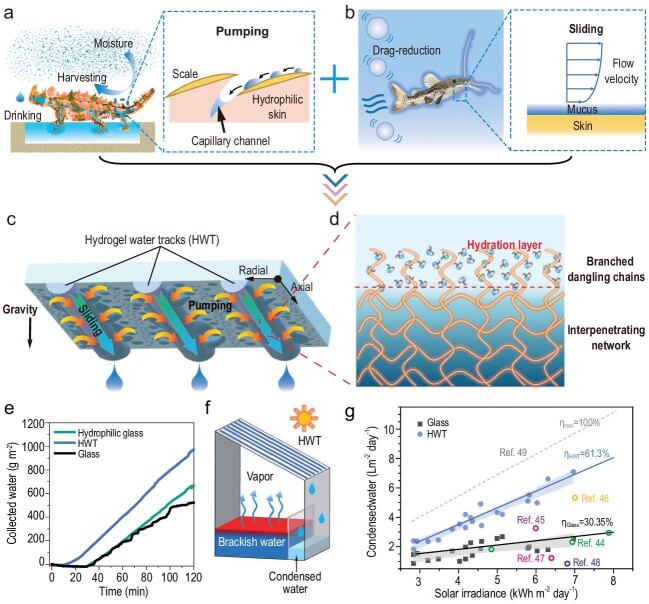
(a) Schematic of continuous water capture and directional water movement by moisture-harvesting lizards enabled by the capillary channels between the scales (side length is ∼1 mm). (b) Schematic of the drag-reduction effect of catfish enabled by mucus. (c) Hydrogel water tracks (HWTs) printed on a silicate glass to collect condensed water droplets and regenerate condensing portion. The blue semi-cylinders represent the HWTs (1 mm in diameter), and the yellow arrows indicate the droplet pumping direction. (d) Schematic diagram of the molecular structure of HWTs. (e) Water droplet collection rates on the HWT pattern and bare glass. (f) Illustration of the pilot study of condensed water collection. (g) Condensed water from outdoor solar distillation devices with glass and HWT-printed glass condensing cover. Refs. 44–49 in the figure refers to the reference numbers in Ref. [5].

Zhang *et al*. [5] took an idea from moisture-harvesting lizards and surface-lubricated catfish, where they used patterned hydrogel fibers on glass to mediate droplet destabilization and to create directional movement, leading to sustainable droplet shedding (Fig. 1). In the work, Laplace pressure difference and surface gradients were synchronously induced to effectively pump all the droplets to the fiber and refresh the condensing portion, similar to the spreading of the water over the skin of the lizards (Fig. 1a and c). The localized water on the fiber could be shed easily via low-drag sliding on the mucus-like hydrogel surface (Fig. 1b and d). Unlike the conventional entirely-hydrophilic surface, the geographically separate superhydrophilic fibers were utilized as lubricated tracks, which served to separate and localize condensed droplets and direct them away from the condensing surface. Based on a range of analytical assessments and characterizations, e.g. interfacial spectroscopy, visualization, and simulation, to substantiate the proposed mechanisms, it came as no surprise that, in the absence of any external energy input, this design could lead to an 85.9% higher condensing rate in the lab and collect 109% more water from outdoor-installed solar distillation devices (Fig. 1e and f).

The unique focus of this work on water vapor condensation, a so far largely overlooked factor in the field of solar distillation, makes it special and valuable. In contrast to the common and still popular strategies of pursuing high evaporation rates by various designs, condensation is proven by this work to be able to squeeze a large amount of water, which would otherwise be lost during the cycle. The principle proposed in this work is versatile and could be incorporated into various evaporative water purification techniques in order to promote water production performance.
